# Supplementary material for: Molecular competition induced Janus hydrogel bioelectronic interface for electroceutical modulation
Source: Nat Commun. 2025 Dec 7;17:455. doi: 10.1038/s41467-025-67143-3 (PMC12800210; doi:10.1038/s41467-025-67143-3)
Supplement: Supplementary file 1 — Supplementary Information [file 41467_2025_67143_MOESM1_ESM.pdf]

Supporting Information

# Molecular Competition Induced Janus Hydrogel Bioelectronic Interface for Electroceutical Modulation

*Xinyu Qu* (曲心宇),[1,2] *Qian Wang* (王倩),[1]* *Hanjun Sun* (孙涵俊),[1] *Dingli Gan* (甘鼎立),[1] *Youliang Zhu* (朱有亮),[3]* *Zhenhua Ni* (倪振华),[2]* *Xiaochen Dong* (董晓臣)[1,4]*

[1]State Key Laboratory of Flexible Electronics (LoFE) & Institute of Advanced Materials (IAM), School of Flexible Electronics (Future Technologies), School of Physical and Mathematical Sciences, Nanjing Tech University (NanjingTech), Nanjing 211816, China.

*E-mail: chelseawq@njtech.edu.cn*

[2]School of Physics and Key Laboratory of Quantum Materials and Devices of Ministry of Education, Southeast University, Nanjing 211189, China

*E-mail: zhni@seu.edu.cn*

[3]State Key Laboratory of Supramolecular Structure and Materials, Institute of Theoretical Chemistry, College of Chemistry, Jilin University, Changchun 130012, China.

*E-mail: youliangzhu@jlu.edu.cn*

[4]School of Chemistry & Materials Science, Jiangsu Normal University, Xuzhou 221116, China.

*E-mail: iamxcdong@njtech.edu.cn*

**Coarse-grained molecular dynamics (CG-MD) simulation**

To reach a large temporal and spatial simulation scale, we performed molecular dynamics (MD) simulations with coarse-grained (CG) models, which took a group of atoms as a bead (Supplementary Fig. S19). Specifically, a monomer of AM is coarse-grained into a *Na* bead and a monomer of SBMA is represented by the three connected beads of *Na1-Q0-Qa* named from left to right. A cluster of four water molecules is represented by a *P4* bead. The bead-spring model with Martini force field methodology is employed in this CG-MD study[1]. In CG-MD simulations, the reduced units of length, energy, mass, and time were $\sigma_0$ = 1.0 nm, $\epsilon_0$ = 1.0 kJ·mol$^{-1}$, $m_0$ = 1.0 amu, and $\tau_0$ = 1.0 ps, respectively. The non-bonded interactions between CG beads were described by Lennard-Jones (LJ) potential and Coulomb potential:

$$V_{\mathrm{LJ}}(r) = 4\epsilon_{ij}\left[\left(\frac{\sigma_{ij}}{r_{ij}}\right)^{12} - \left(\frac{\sigma_{ij}}{r_{ij}}\right)^{6}\right] \qquad \text{(S1)}$$

$$V_{Coulomb}(r) = f\,\frac{q_i q_j}{\varepsilon_r r} \qquad \text{(S2)}$$

where $r_{ij}$ is the distance between bead $i$ and $j$, the interaction parameters $\epsilon_{ij}$ and $\sigma_{ij}$ are listed in Supplementary Table S1, and $f=\frac{1}{4\pi\varepsilon_0}=$ 138.9 kJ·mol$^{-1}$·nm·e$^{-2}$. We note that both potentials were truncated at $r_{cut}$ = 1.2 $\sigma_0$ and the Coulomb potential was smooth from 0.9 to 1.2 $\sigma_0$ to ensure the zeroing of force and energy at the truncation radius. The relative dielectric constant $\varepsilon_r$ is set to be 15.0. The *Q0* bead and *Qa* bead carry a positive charge and a negative charge, respectively. The harmonic potential was employed to describe bond stretching interactions in the bead-spring model:

$$V_{\mathrm{bond}}(r) = \frac{1}{2}K_{\mathrm{bond}}(r - r_0)^2 \qquad \text{(S3)}$$

where $r_0$ = 0.47 $\sigma_0$ and $K_{\mathrm{bond}}$ = 1250.0 $\epsilon_0/\sigma_0^2$.

The simulation system was composed of 484,000 AM monomers, 240,000 SBMA monomers,

and 1,572,000 water molecules (52,4000 CG beads), totaling 1,728,000 CG beads, in a box of $30.0\times60.0\times120.0\ \sigma_0^3$. The CG-MD simulations were performed with a time step of 0.02 $\tau_0$, under canonical ensemble conditions with T = 300 K. To simulate the polymerization process, we employed a polymerization model with 4,000 initiators in the bottom of simulation box[2,3]. The polymerization process was simulated for $8.0\times10^7$ time steps (1.6 μs) after the equilibrium simulation of $1.0\times10^7$ time steps (0.2 μs), with the reaction probabilities $Pr_{Na\text{-}Na}$ = 0.01, $Pr_{Na1\text{-}Na1}$ = 0.001, and $Pr_{Na\text{-}Na1}$ = 0.001. The formed gel structure (Supplementary Fig. S20) after polymerization simulation was used for structural analysis and further tensile test simulation. In particular, we performed isovolumic tensile tests with the box length in the Z-direction increasing linearly from $120\sigma_0$ to $240\sigma_0$, while the *X*- and *Y*-direction lengths contracted proportionally to maintain constant volume. The simulations were performed using the in-house developed software on a GPU[4,5].

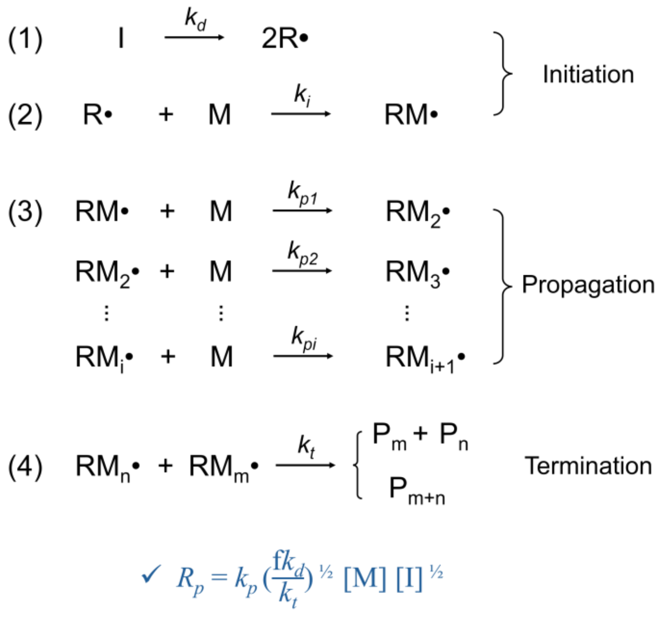


**Fig. S1.** Free radical polymerization kinetics. I: initiator; R•: primary radical; M: monomer; RM•: monomer radical; $k_d$: decomposition rate constant; $k_p$: propagation rate constant; $k_t$: termination rate constant; f: initiation efficiency.

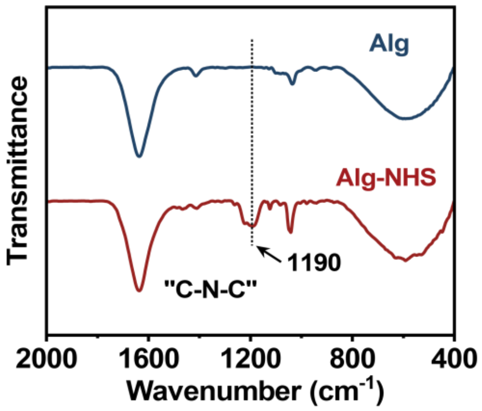


**Fig. S2.** FT-IR spectrum of Alg and Alg-NHS.

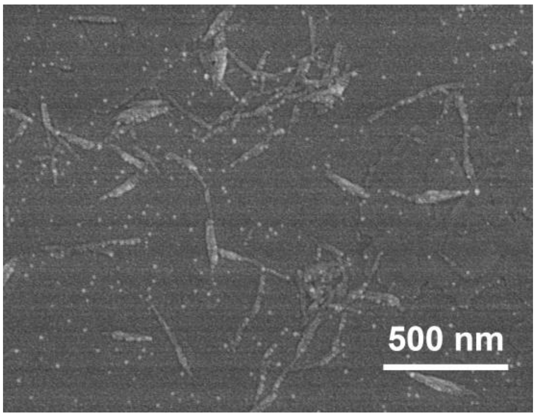


**Fig. S3.** SEM image of CNCs.

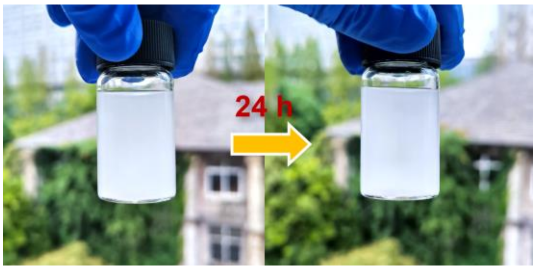


**Fig. S4.** Stable dispersion of CNCs in aqueous solution for 24 h.

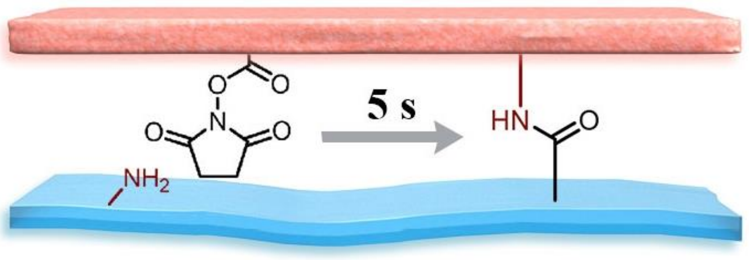


**Fig. S5.** The process of establishing covalent adhesion bonds by NHS ester groups.

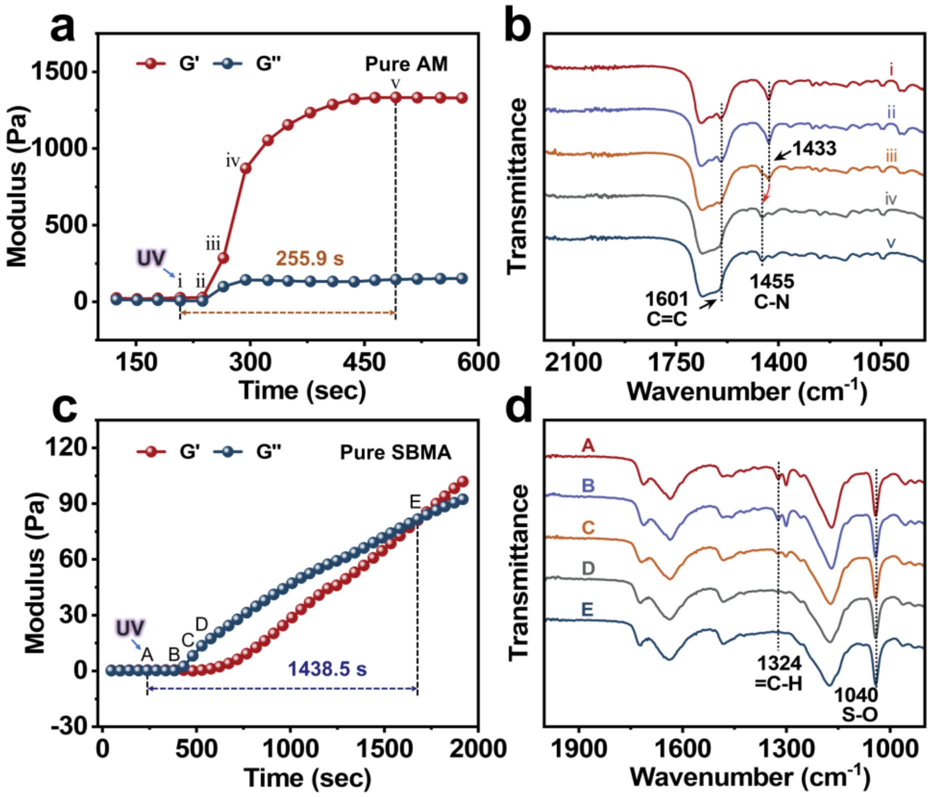


**Fig. S6.** Rheology and corresponding FT-IR of pure AM and pure SBMA during gelation progress.

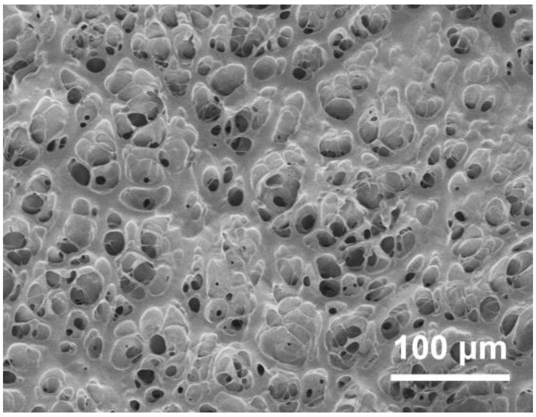


**Fig. S7.** SEM image of the hydrogel synthesized by conventional thermal initiation method.

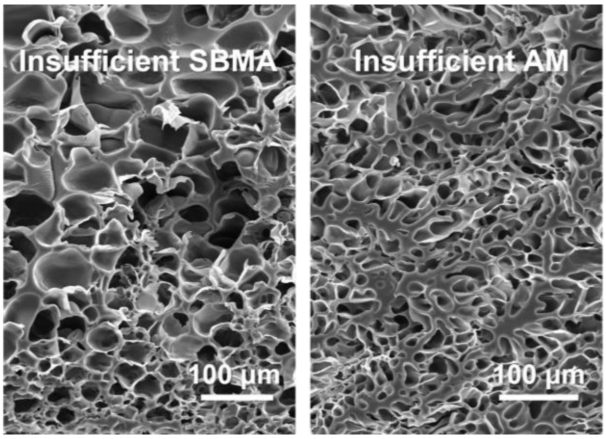


**Fig. S8.** Cross-sectional SEM images of hydrogel network with insufficient content of SBMA and AM.

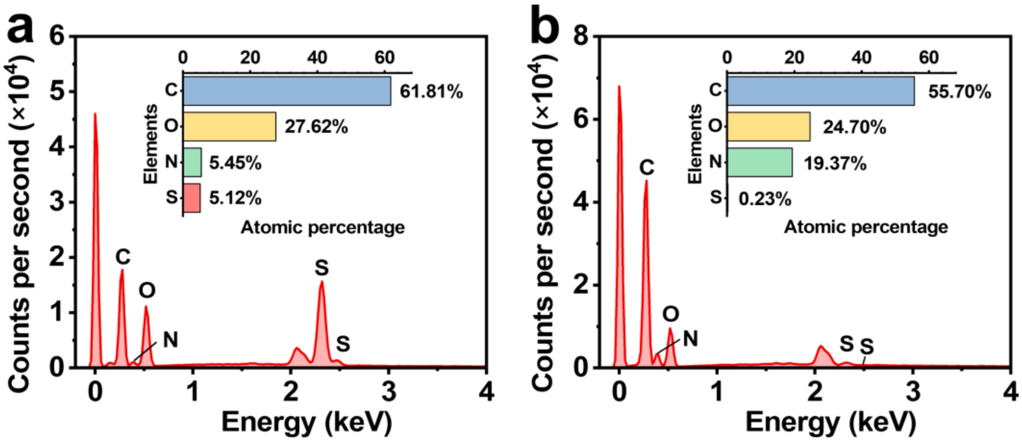


**Fig. S9.** (a, b) Element proportional analysis of adhesive and non-adhesive sides.

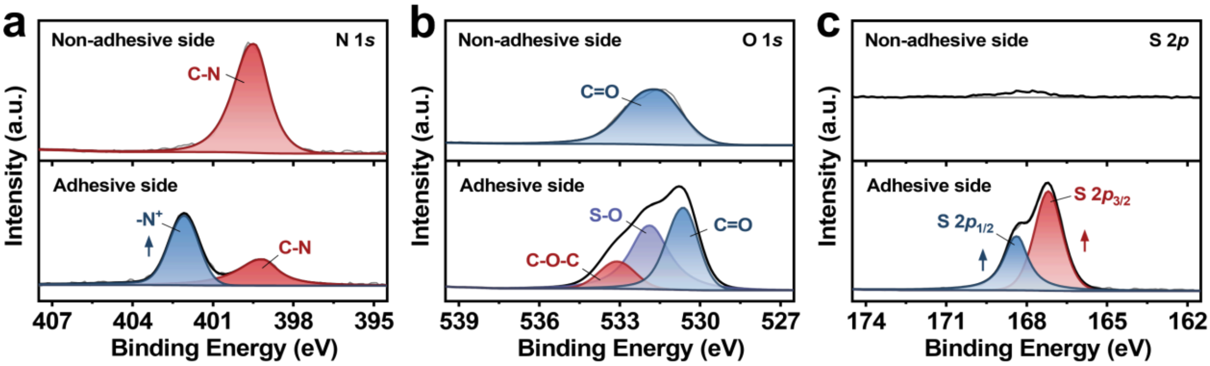


**Fig. S10.** Peak-fitting XPS spectra of adhesive and non-adhesive sides.

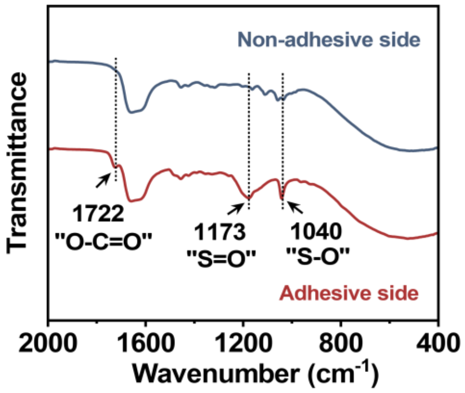


**Fig. S11.** FT-IR spectra of adhesive and non-adhesive sides of Janus hydrogel without CNCs/ALG-NHS.

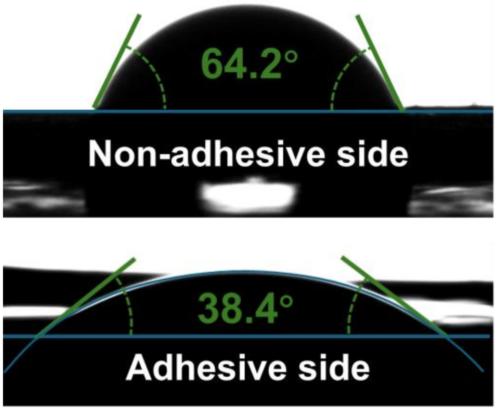


**Fig. S12.** Contact angle of adhesive and non-adhesive side.

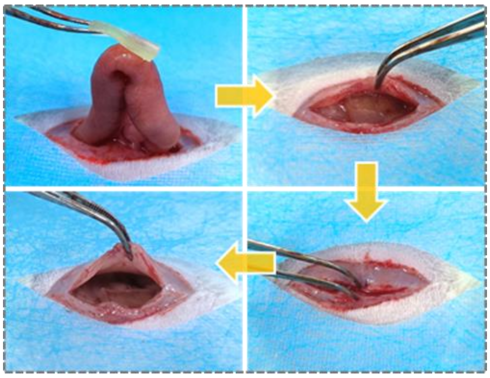

**Fig. S13.** The unilateral adhesion of Janus hydrogel.

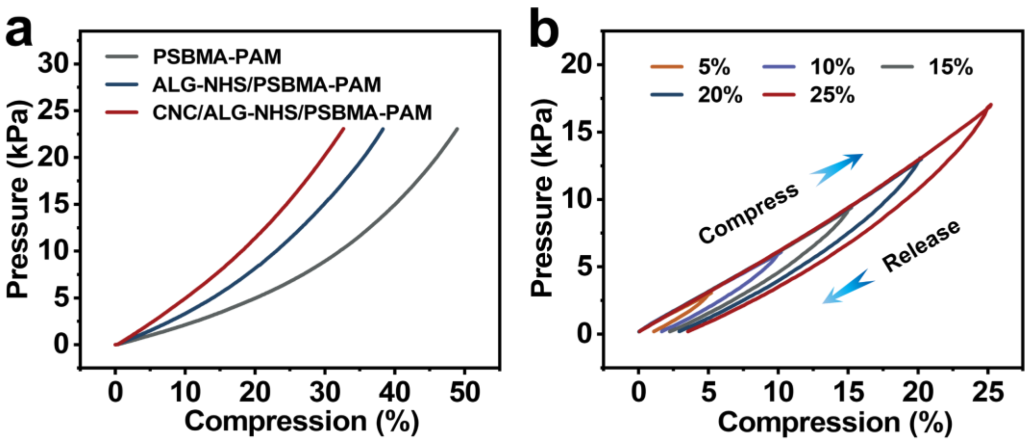


**Fig. S14.** (a) Compressive/tensile stress-strain curves of different components. (b) Compressive loading-recovery cycles.

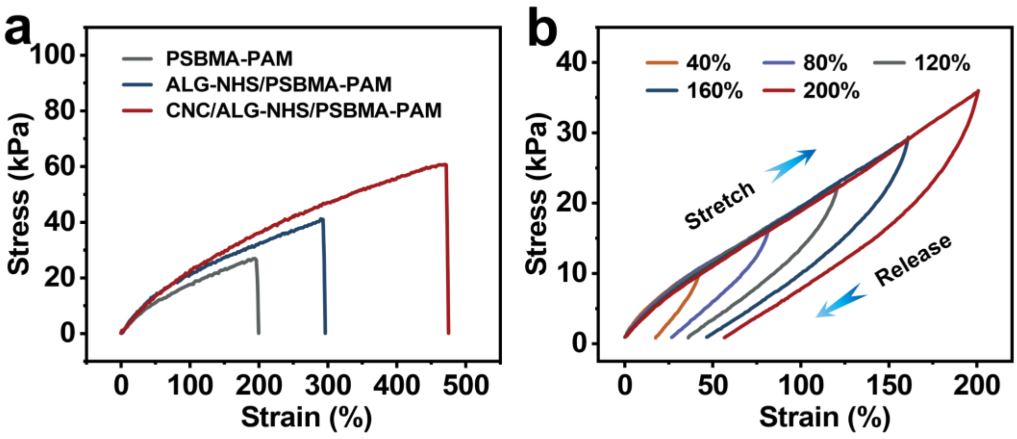


**Fig. S15.** (a) Tensile stress-strain curves of different components. (b) Tensile loading-recovery cycles.

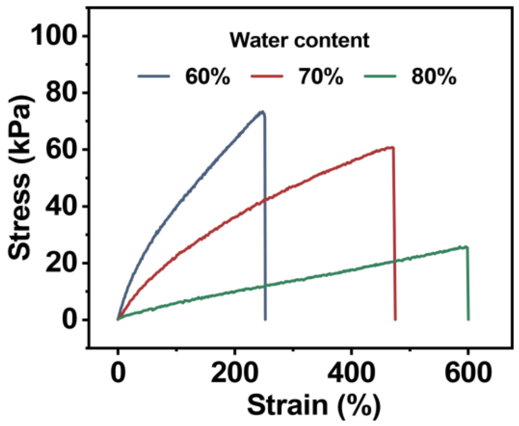


**Fig. S16.** (a) Compressive/tensile stress-strain curves of different water contents.

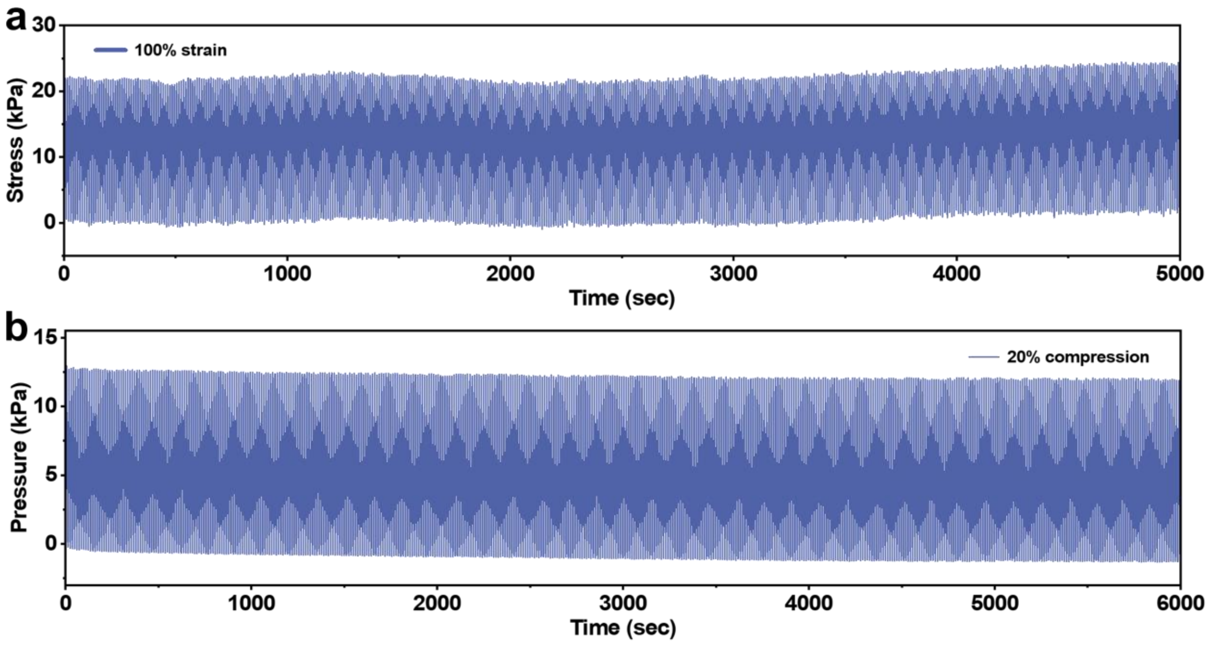


**Fig. S17.** (a, b) Tensile/compressive cycle stability curve.

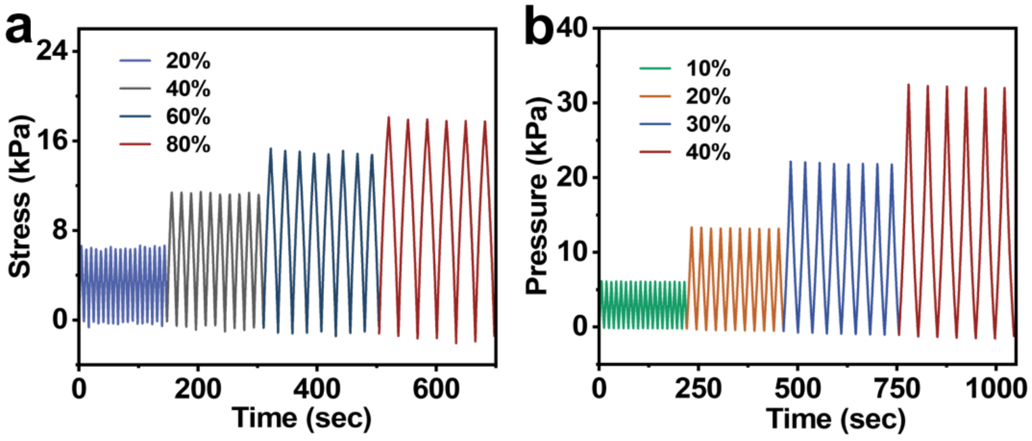


**Fig. S18.** Cyclic tensile/compressive curves at different strains.

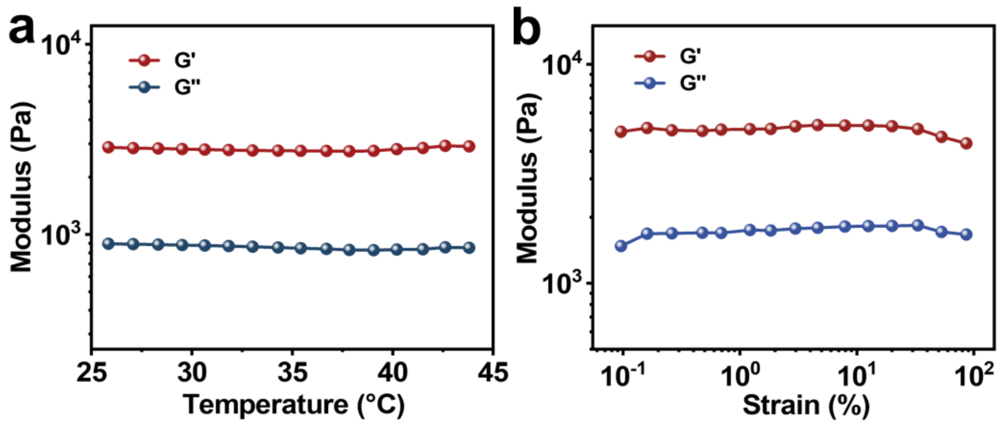


**Fig. S19.** (a, b) Rheological properties of *G′* and *G″* values at different temperatures and strains.

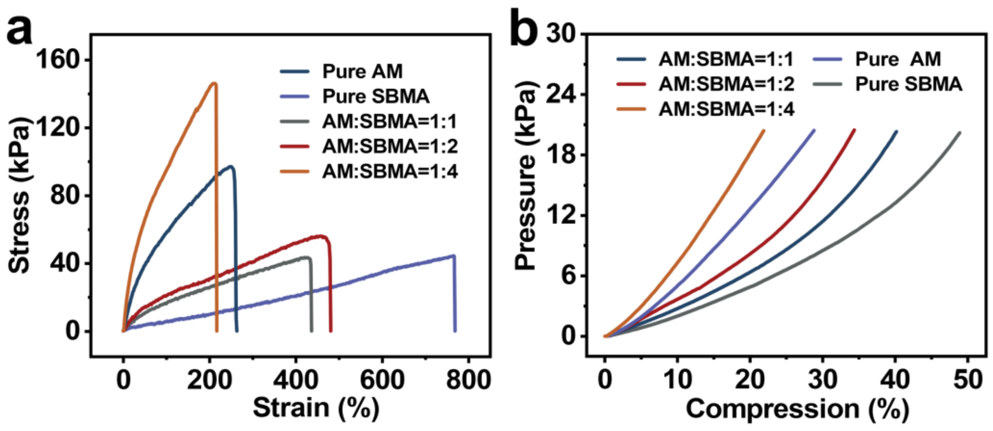


**Fig. S20.** (a, b) Tensile stress-strain curves at different contents.

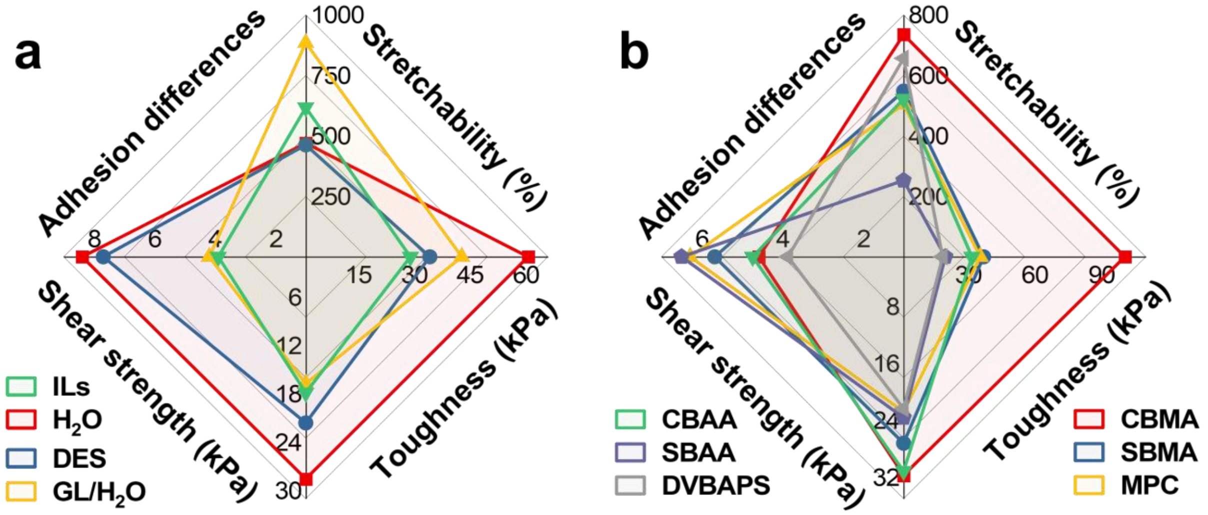


**Fig. S21.** (a) Comparison of the properties of Janus hydrogels at different solvents. (ILs: Ionic liquids; DES: deep eutectic solvent; GL: glycerol) (b) Comparison of the properties of Janus hydrogels at acrylic acid (AA) and different zwitterionic species.

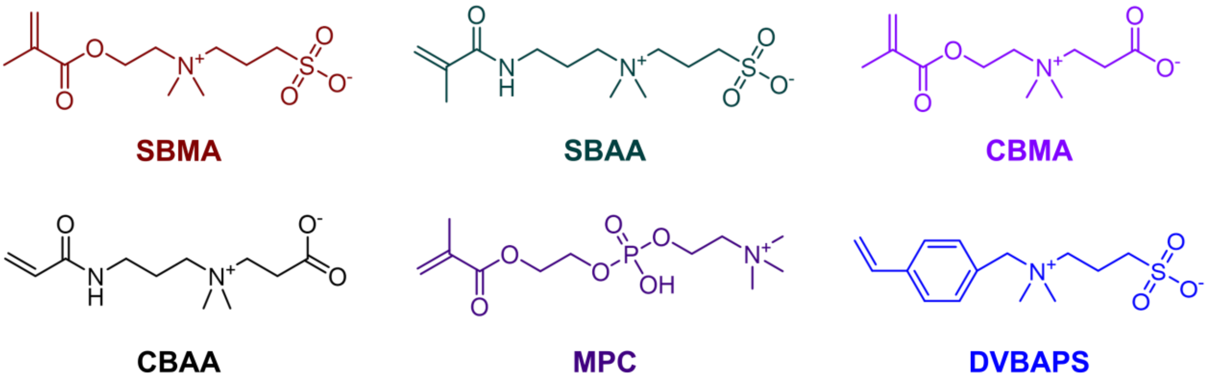


**Fig. S22.** Different vinyl zwitterionic monomers.

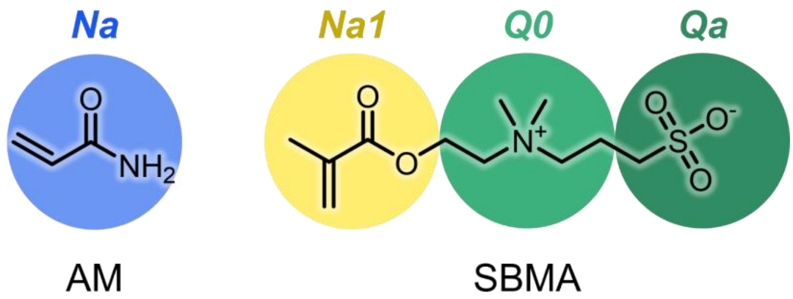


**Fig. S23.** Color-mapped modeling results of AM and SBMA.

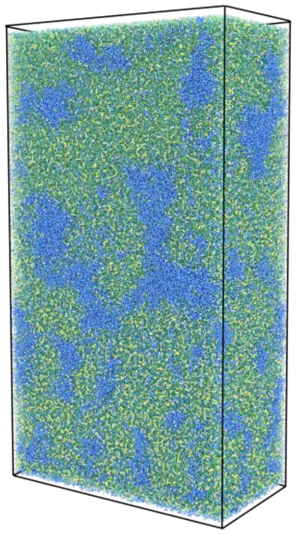

**Fig. S24.** Molecular dynamics model of Janus hydrogel.

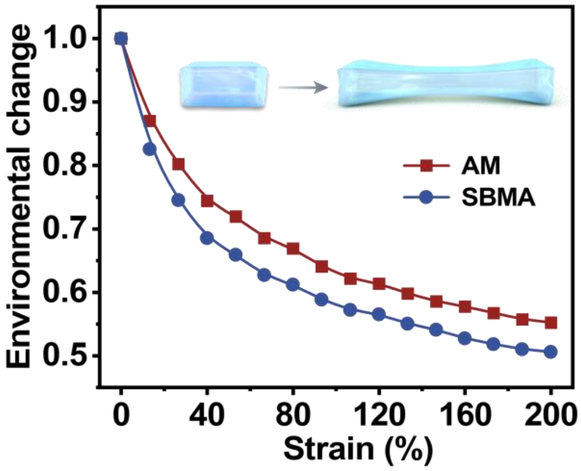


**Fig. S25.** Environmental change under different strains.

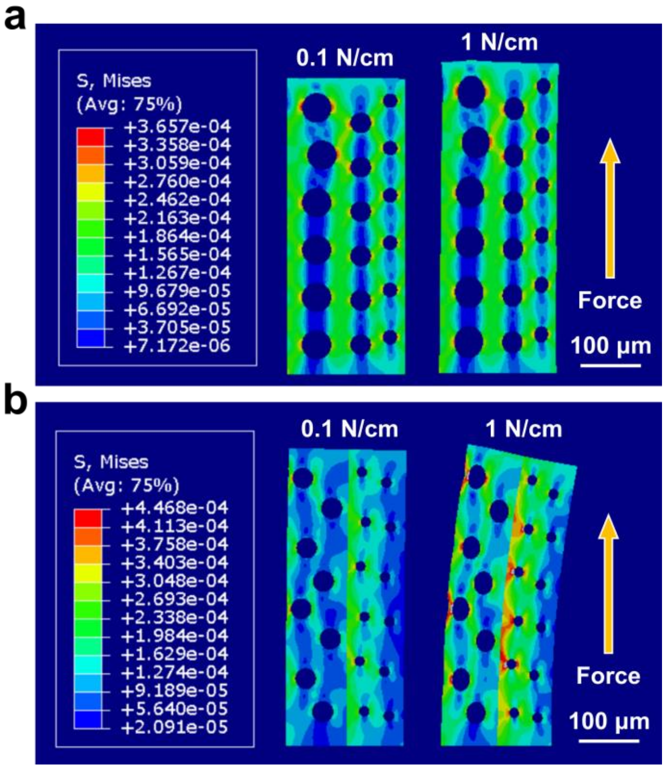


**Fig. S26.** FEA of gradient structure and bilayer structure under stretching.

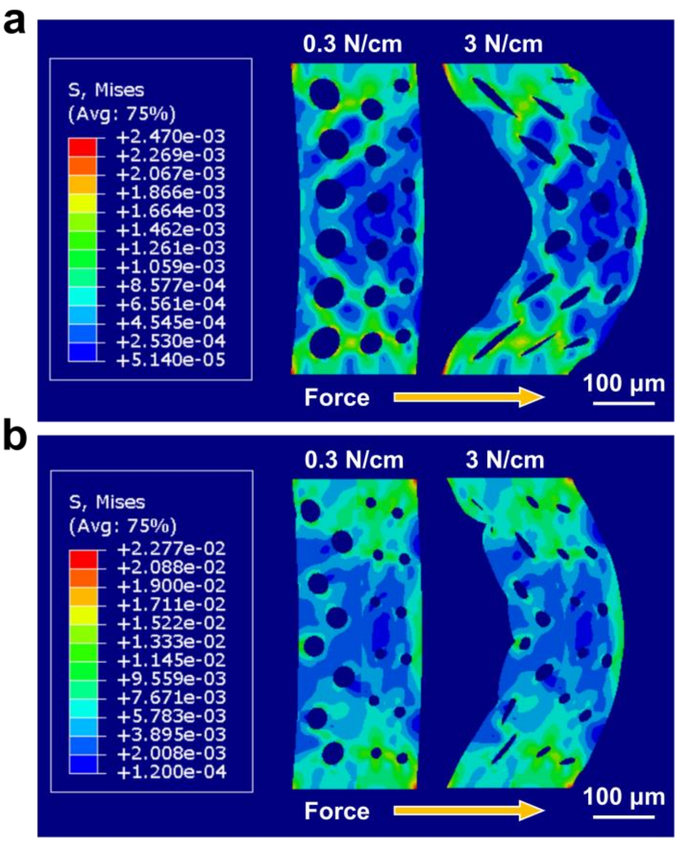


**Fig. S27.** FEA of gradient structure and bilayer structure under bending.

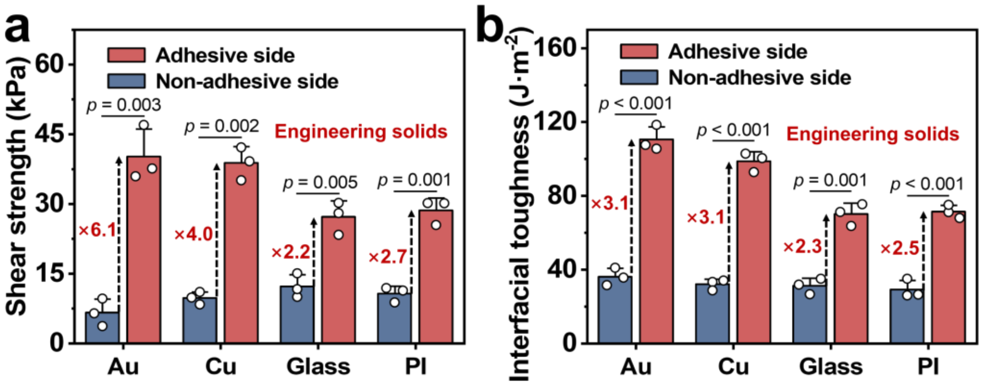


**Fig. S28.** (a, b) Shear strength and interfacial toughness curves with different engineered solids (n = 3 independent samples).

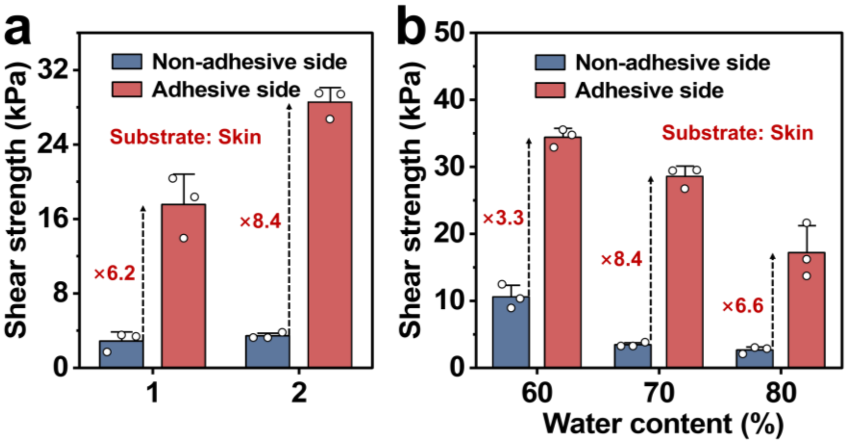


**Fig. S29.** (a) Shear strength at different components (n = 3 independent samples). (1: w/o CNCs/ALG-NHS; 2: w/ CNCs/ALG-NHS) (b) Shear strength at different water contents (n = 3 independent samples).

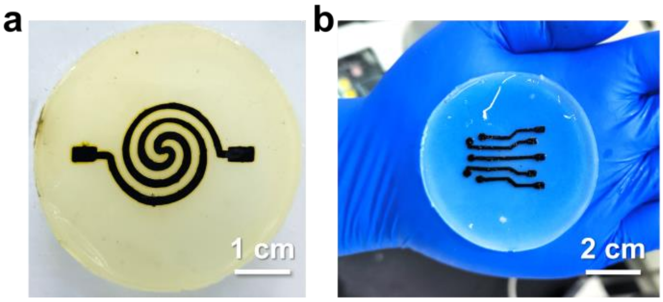


**Fig. S30.** (a) Images of tree-ring-like spiral-patterned hydrogel interface. (b) Images of a multi-channel hydrogel interface.

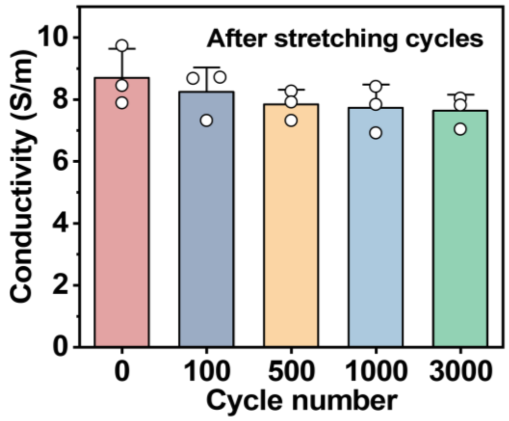


**Fig. S31.** Conductivity of the hydrogel interface at different stretching cycles (100% strain) (n = 3 independent samples).

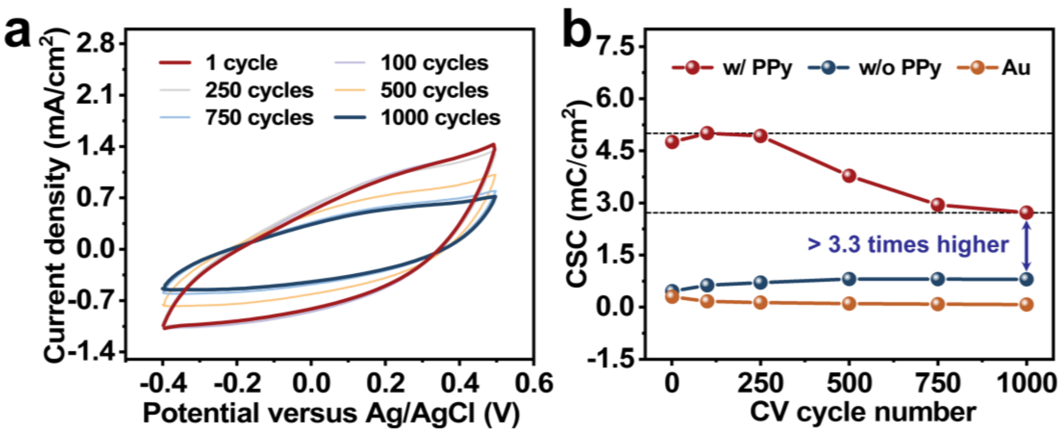


**Fig. S32.** (a) CV curves of Janus hydrogel at different charge-discharge cycles. (b) CSC value at different CV cycles.

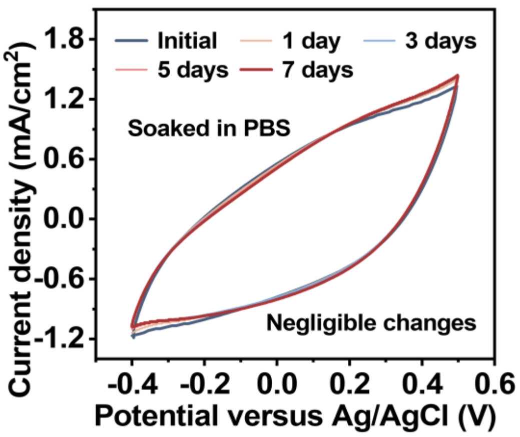


**Fig. S33.** CV curves after soaking in PBS solution for different days.

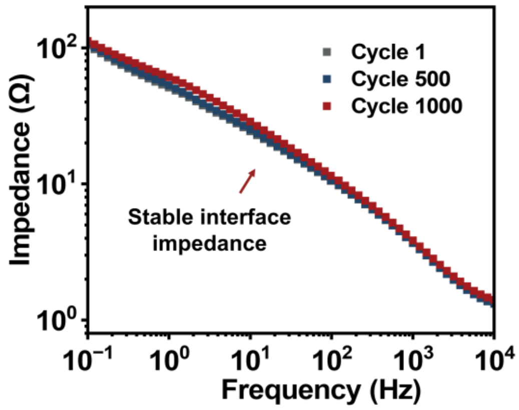


**Fig. S34.** Impedance at different stretching cycles.

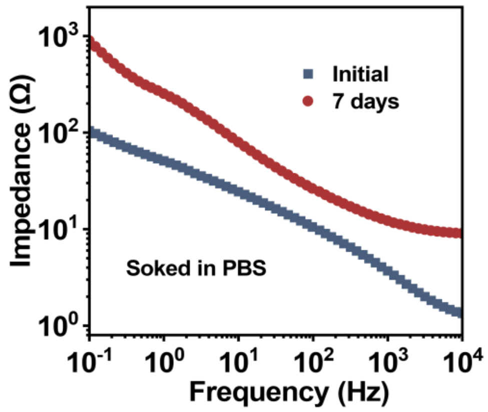


**Fig. 35.** Interfacial impedance after soaking in PBS solution for different days.

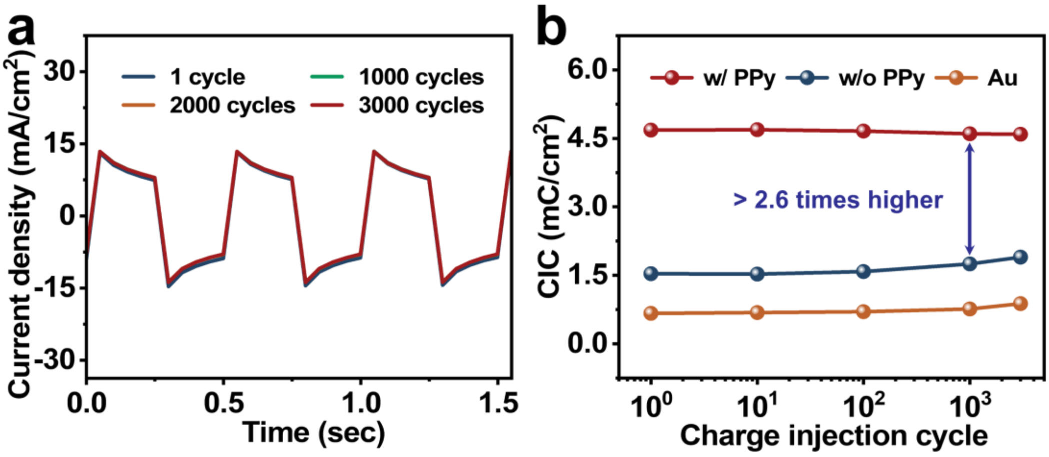


**Fig. S36.** (a) Charge injection curves of Janus hydrogel at different charge injection cycles. (b) CIC value at different charge injection cycles.

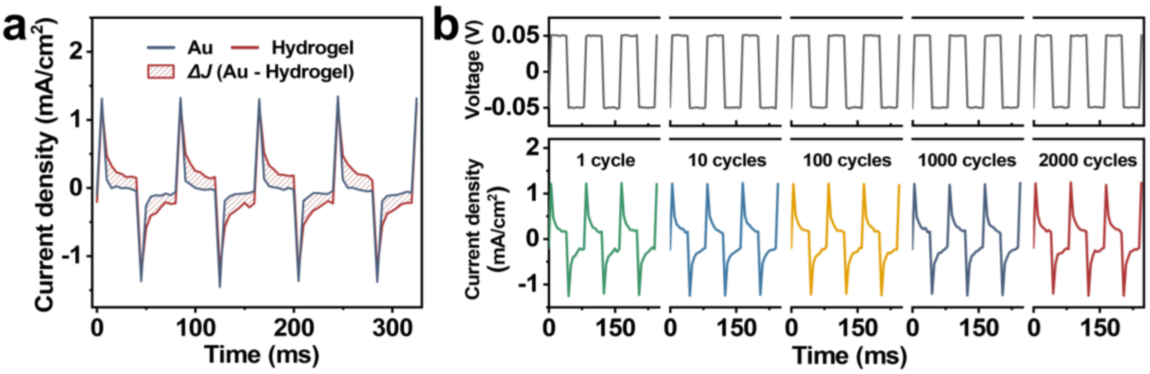


**Fig. 37.** (a, b) Charge injection curves with biphasic pulses of ± 0.05 V for 2000 cycles.

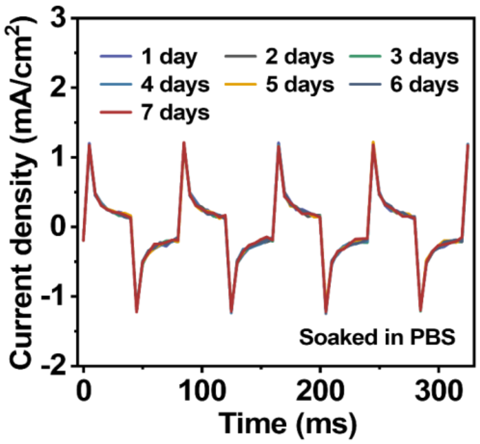


**Fig. S38.** Charge injection curves with biphasic pulses of ± 0.05 V after soaking in PBS solution for different days.

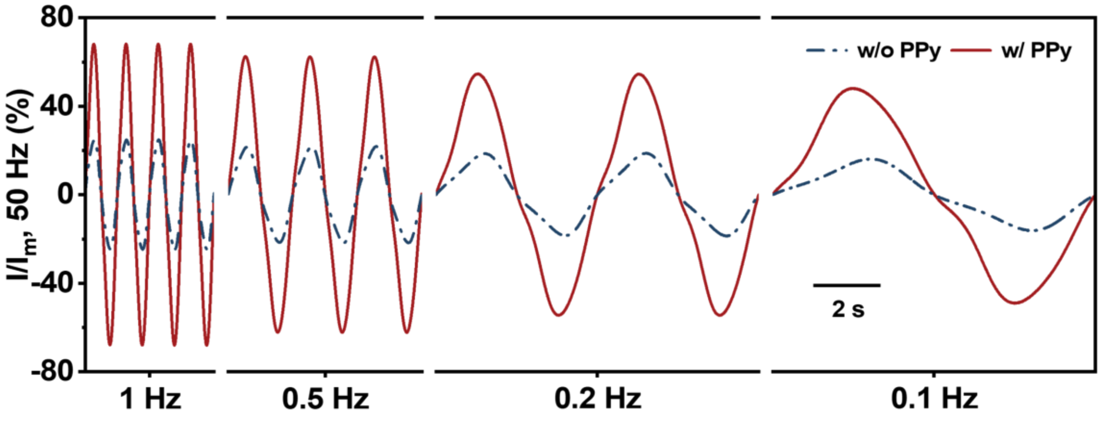


**Fig. S39.** Normalized current *vs.* frequency curves of Janus hydrogel and control groups upon sinusoidal AC voltage.

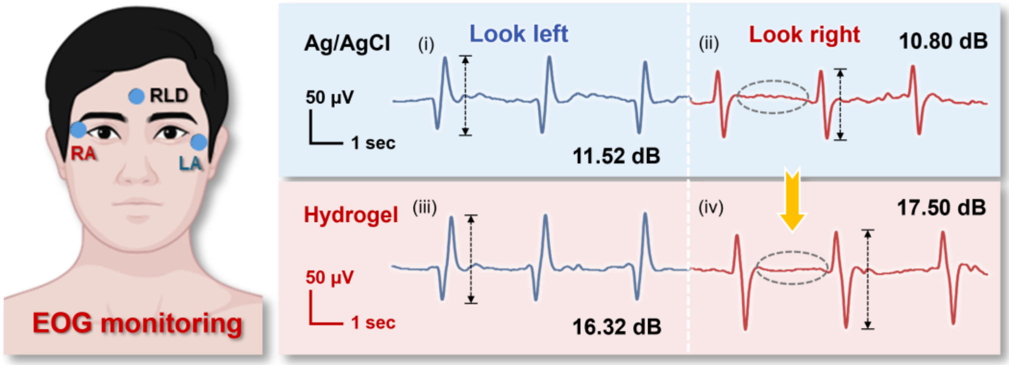


**Fig. S40.** EOG signals captured by commercial Ag/AgCl electrode and Janus hydrogel interface. Created in BioRender. Shao, J. (2025) https://BioRender.com/luci6pc.

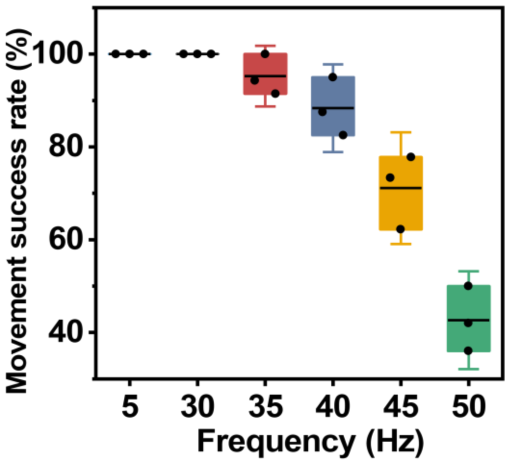


**Fig. S41.** The success rate of leg movement under different stimulus frequencies (n = 3 independent samples).

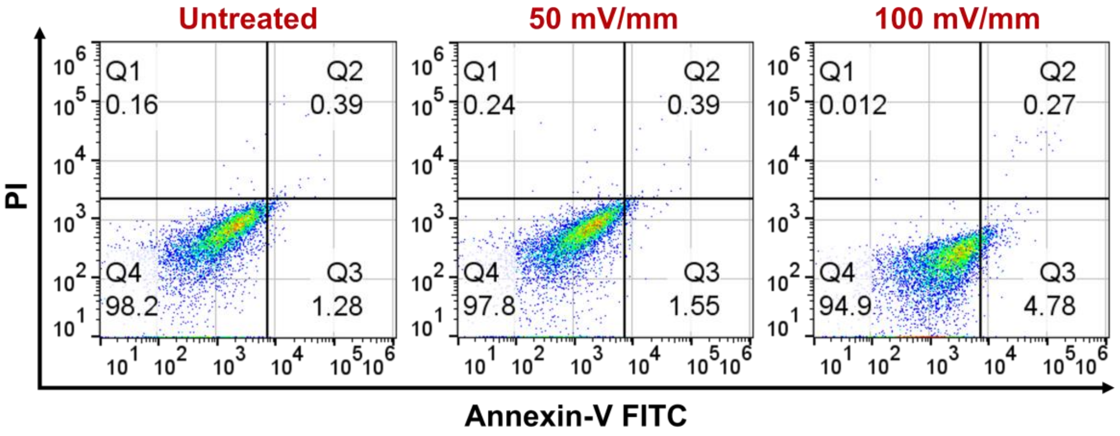


**Fig. S42.** Flow cytometry of cells treated with different voltage intensities.

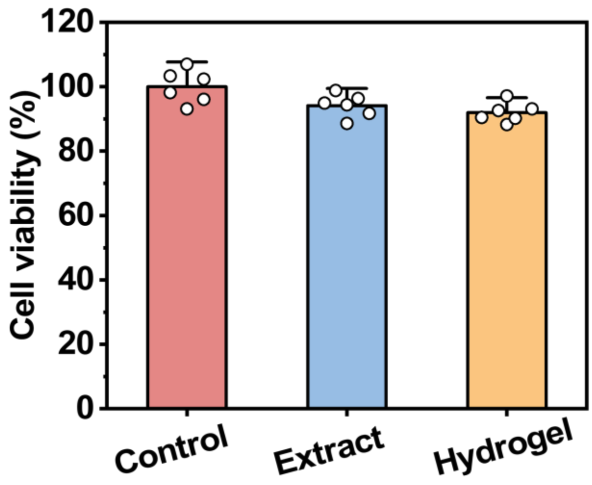


**Fig. S43.** Cell viability of Janus hydrogel (n = 6 independent samples).

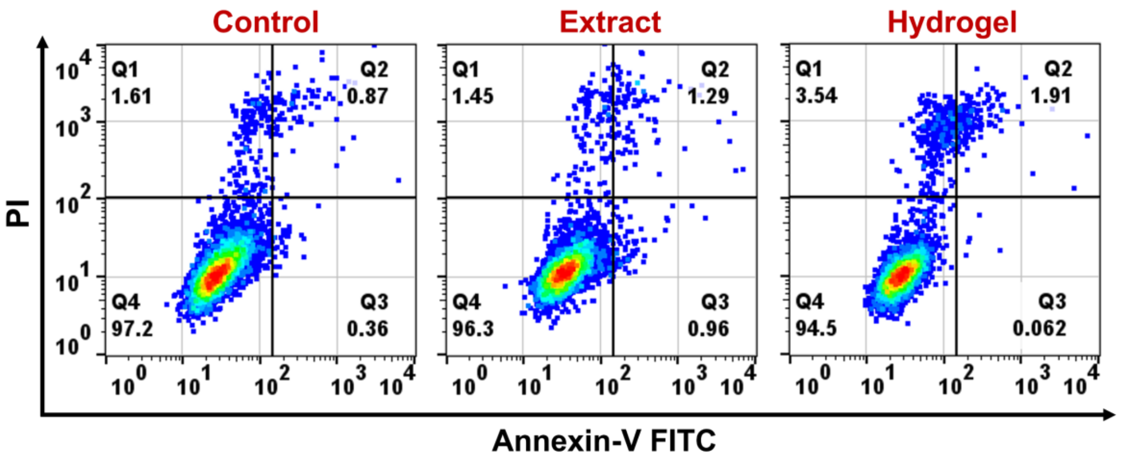


**Fig. S44.** Flow cytometry of cells incubated with Janus hydrogel.

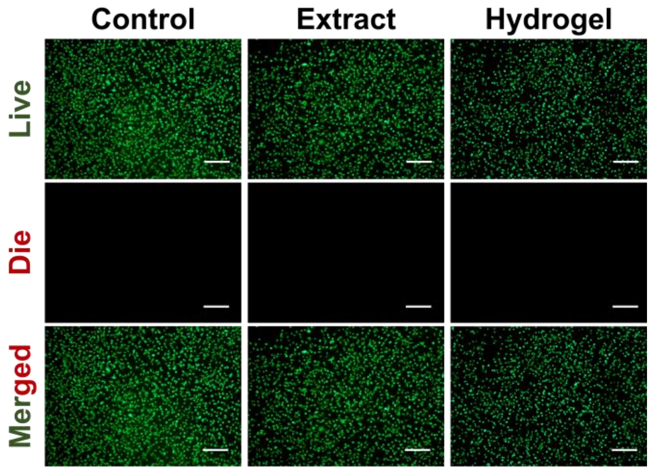


**Fig. S45.** Cell live/death staining of Janus hydrogel. Scale bar: 100 μm.

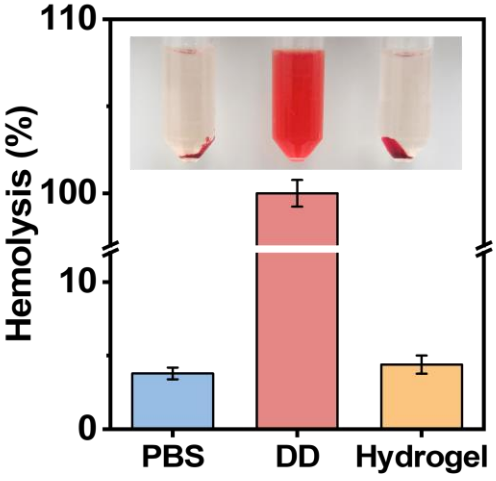


**Fig. S46.** (a) Hemolytic activity of Janus hydrogel (n = 3 independent samples).

| Index | Normal | Hydrogel |
|---|---|---|
| WBC ($10^9$/L) | 7.3±0.17 | 6.33±0.40 |
| RBC ($10^{12}$/L) | 7.38±0.22 | 7.82±1.08 |
| PLT ($10^9$/L) | 1006.33±70.72 | 1060±86.26 |
| HGB (g/L) | 137.33±3.79 | 140.33±9.87 |
| ALT (U/L) | 26.31±0.29 | 26.68±0.13 |
| AST (U/L) | 102.58±0.43 | 102.95±0.73 |
| BUN (mg/dl) | 15.32±0.18 | 15.40±0.11 |
| CREA (µmol/L) | 17.31±0.27 | 16.67±0.06 |

**Fig. S47.** Hematological and blood biochemical analyses after implantation of Janus hydrogel.

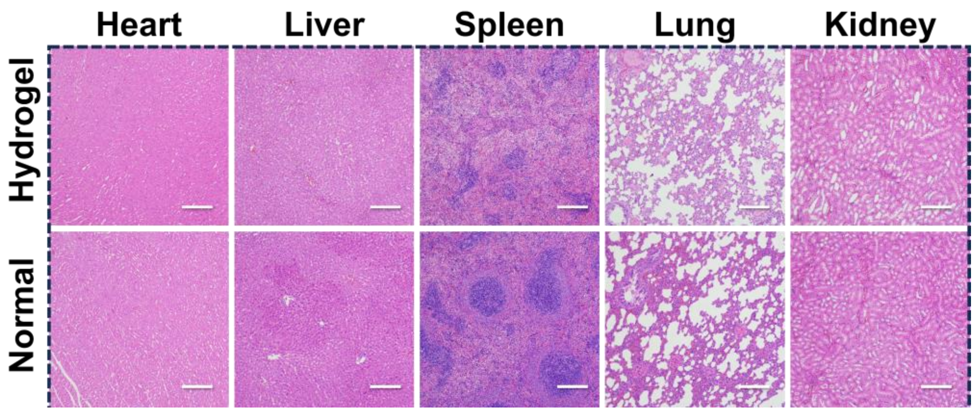


**Fig. S48.** H&E staining histologic section of essential organs. Scale bar: 100 µm.

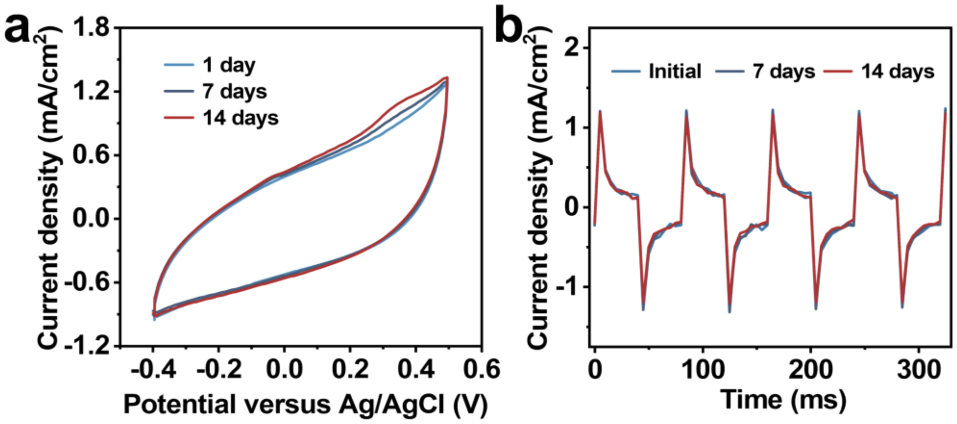


**Fig. S49.** CV and Charge injection curves after subcutaneous implantation for different days.

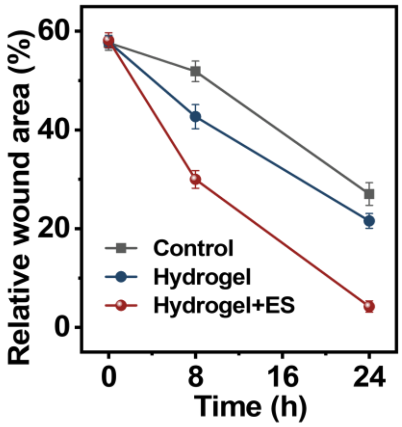


**Fig. S50.** Statistical analysis of relative wound area in the scratch assay.

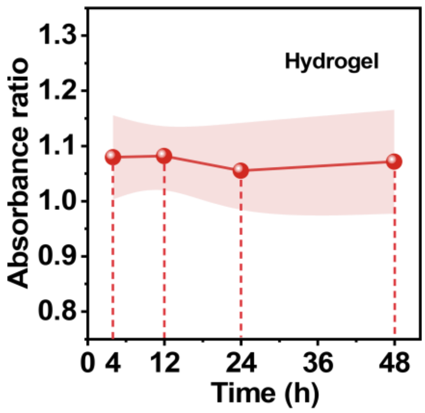


**Fig. S51.** Absorbance ratio of cells in the hydrogel group to those in the control group by CCK-8 assay. Color bars indicate standard deviations.

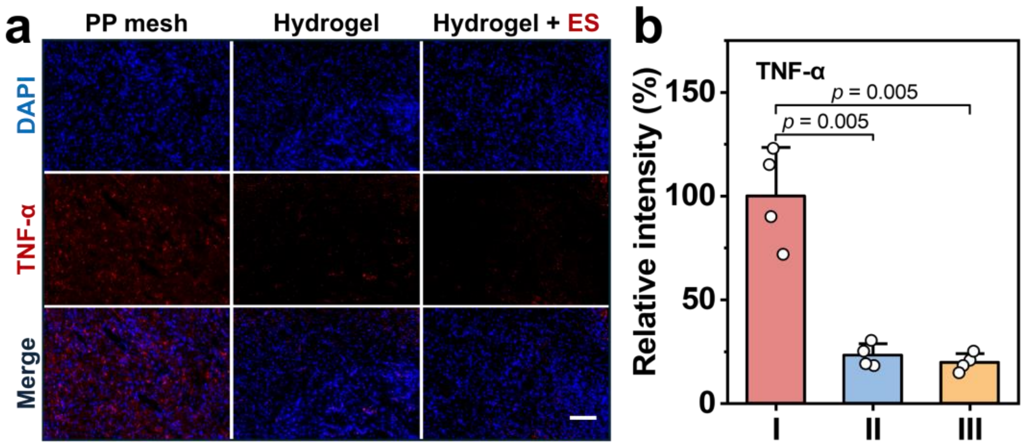


**Fig. S52.** (a) Representative fluorescence images of TNF-$\alpha$ (red) in the tissues. Scale bar: 100 μm. (b) Fluorescence intensity of TNF-$\alpha$ in the tissues (n = 4 independent samples).

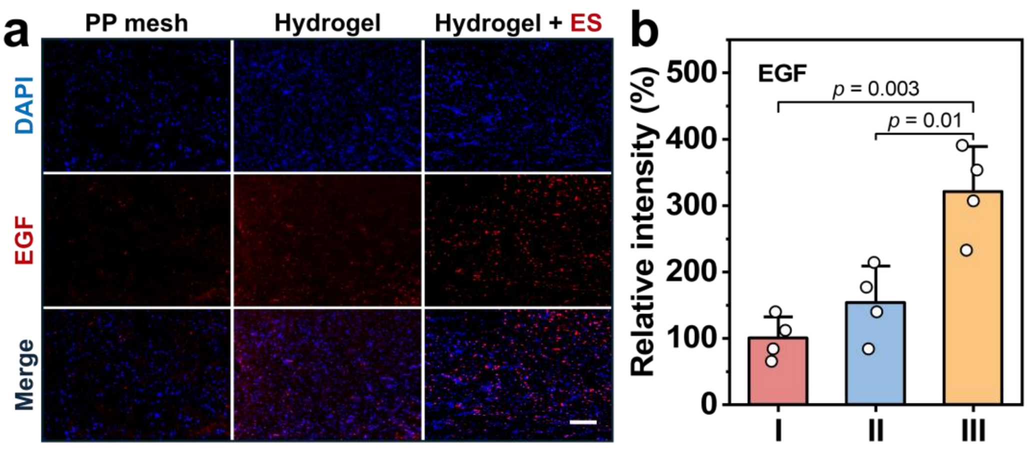


**Fig. S53.** (a) Representative fluorescence images of EGF (red) in the tissues. Scale bar: 100 μm. (b) Fluorescence intensity of EGF in the tissues (n = 4 independent samples).

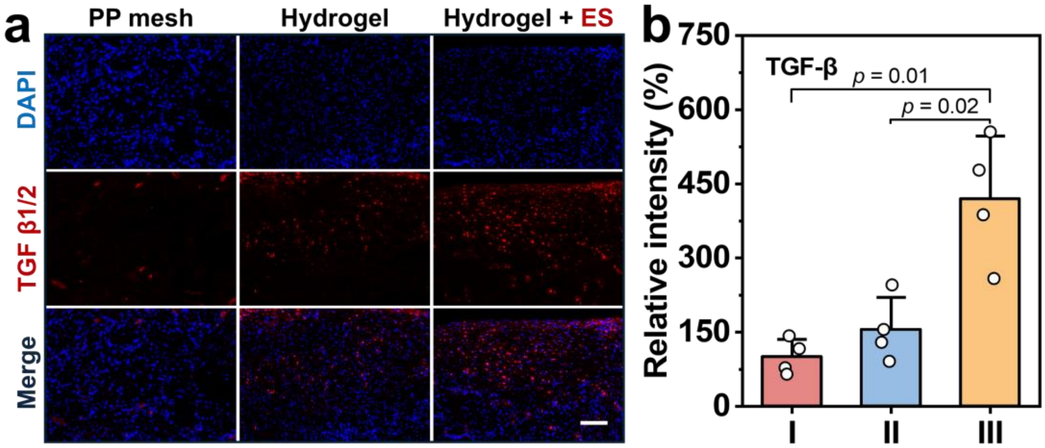


**Fig. S54.** (a) Representative fluorescence images of TGF-$\beta$ (red) in the tissues. Scale bar: 100 µm. (b) Fluorescence intensity of TGF-$\beta$ in the tissues (n = 4 independent samples).

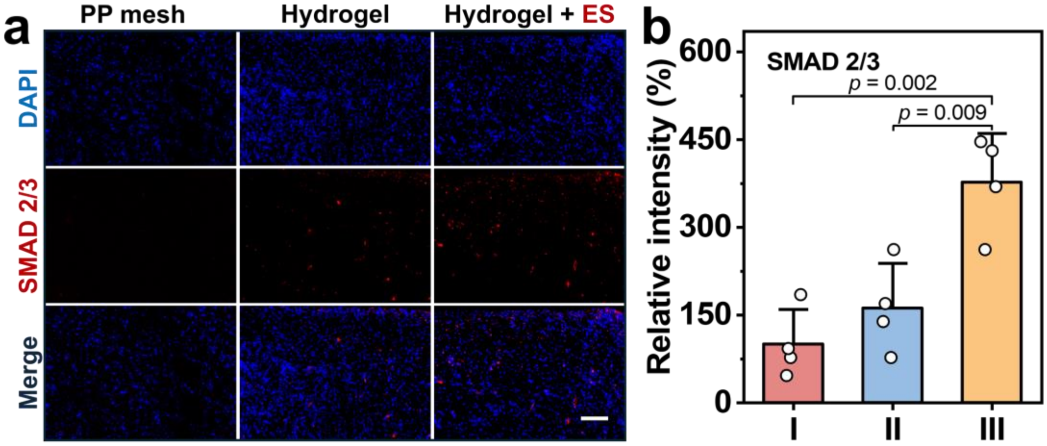


**Fig. S55.** (a) Representative fluorescence images of SMAD 2/3 (red) in the tissues. Scale bar: 100 µm. (b) Fluorescence intensity of SMAD 2/3 in the tissues (n = 4 independent samples).

**Table S1** The parameters of LJ interactions.

| $\epsilon_{ij}/\sigma_{ij}$ | ***Na*** | ***Na1*** | ***Q0*** | ***Qa*** | ***P4*** |
|---|---|---|---|---|---|
| ***Na*** | $4.0\epsilon_0/0.47\sigma_0$ | $4.0\epsilon_0/0.47\sigma_0$ | $4.0\epsilon_0/0.47\sigma_0$ | $4.0\epsilon_0/0.47\sigma_0$ | $4.3\epsilon_0/0.47\sigma_0$ |
| ***Na1*** | | $4.0\epsilon_0/0.47\sigma_0$ | $4.0\epsilon_0/0.47\sigma_0$ | $4.0\epsilon_0/0.47\sigma_0$ | $4.3\epsilon_0/0.47\sigma_0$ |
| ***Q0*** | | | $3.5\epsilon_0/0.47\sigma_0$ | $4.5\epsilon_0/0.47\sigma_0$ | $5.6\epsilon_0/0.47\sigma_0$ |
| ***Qa*** | | | | $5.0\epsilon_0/0.47\sigma_0$ | $5.6\epsilon_0/0.47\sigma_0$ |
| ***P4*** | | | | | $5.0\epsilon_0/0.47\sigma_0$ |

## References


1. Marrink S., Risselada H., Yefimov S., Tieleman D. & Vries A. The MARTINI force field: Coarse grained model for biomolecular simulations. *J. Phys. Chem. B* **111**, 7812-7824 (2007).
2. Liu H., Zhu Y., Lu Z. & Müller-Plathe F. A kinetic chain growth algorithm in coarse-grained simulations. *J. Comput. Chem.* **37**, 2634-2646 (2016).
3. Zhu Y., *et al.* Mechanisms of defect correction by reversible chemistries in covalent organic frameworks. *J. Phys. Chem. Lett.* **11**, 9952-9956 (2020).
4. Zhu Y., *et al.* GALAMOST: GPU-accelerated large-scale molecular simulation toolkit. *J. Comput. Chem.* **34**, 2197-2211 (2013).
5. Zhu Y., *et al.* Employing multi-GPU power for molecular dynamics simulation: an extension of GALAMOST. *Mol. Phys.* **116**, 1065-1077 (2018).
