# Supplementary material for: Molecular competition induced Janus hydrogel bioelectronic interface for electroceutical modulation
Source: Nat Commun. 2025 Dec 7;17:455. doi: 10.1038/s41467-025-67143-3 (PMC12800210; doi:10.1038/s41467-025-67143-3)
Supplement: Supplementary file 2 — Description of Additional Supplementary Information [file 41467_2025_67143_MOESM2_ESM.pdf]

Description of Additional Supplementary Information

Supplementary Movie 1: Representative burst pressure test process of the Janus hydrogel.
